# Prognostic Impact of Statins in Heart Failure with Preserved Ejection Fraction

**DOI:** 10.3390/jcm13195844

**Published:** 2024-09-30

**Authors:** Samanta Ortega-Hernández, Sonia González-Sosa, Alicia Conde-Martel, Joan Carles Trullàs, Pau Llàcer, José Pérez-Silvestre, José Carlos Arévalo-Lorido, Jesús Casado, Francesc Formiga, Luis Manzano, Noel Lorenzo-Villalba, Manuel Montero-Pérez-Barquero

**Affiliations:** 1 Internal Medicine Department, Hospital Universitario de Gran Canaria Dr. Negrín, 35010 Las Palmas de Gran Canaria, Las Palmas, Spain; samanta.ortega101@alu.ulpgc.es (S.O.-H.);; 2 Health Sciences Faculty, Universidad de Las Palmas de Gran Canaria, 35016 Las Palmas, Spain; 3 Internal Medicine Department, Hospital d’Olot i Comarcal de la Garrotxa, 17800 Girona, Spain; jctv5153@comg.cat; 4 Tissue Repair and Regeneration Laboratory (TR2Lab), Institut de Recerca i Innovació en Ciències de la Vida i de la Salut a la Catalunya Central (IrisCC), Faculty of Medicine, University of Vic—Central University of Catalonia (UVic-UCC), Ctra. de Roda, 70, 08500 Vic, Barcelona, Spain; 5 Internal Medicine Department, Hospital Universitario Ramón y Cajal, Instituto Ramón y Cajal de Investigación Sanitaria (IRYCIS), Universidad de Alcalá, 28801 Madrid, Spain; 6 Internal Medicine Department, Consorcio Hospital General Universitario de Valencia, 46014 Valencia, Spain; 7 FundeSalud, 06800 Mérida, Badajoz, Spain; 8 Internal Medicine Department, Hospital Universitario de Getafe, 28905 Madrid, Spain; 9 Facultad de Ciencias Biomédicas y de la Salud, Universidad Europea de Madrid, 28670 Madrid, Spain; 10 Internal Medicine Department, Hospital Universitario Bellvitge, 08907 L’Hospitalet de Llobregat, Barcelona, Spain; 11 Service de Médecine Interne, Hôpitaux Universitaires de Strasbourg, 67000 Strasbourg, France; 12 Internal Medicine Department, Hospital Universitario Reina Sofía, 14004 Córdoba, Spain; montero.manolo@gmail.com

**Keywords:** heart failure, preserved ejection fraction, mortality, statins

## Abstract

**Background:** Heart failure (HF) with preserved ejection fraction (pEF) has lacked effective treatments for reducing mortality. However, previous studies have found an association between statin use and decreased mortality in patients with HFpEF. The aim of this study was to analyse whether statin therapy is associated with a reduction in mortality in these patients and whether the effect differs according to the presence or absence of ischaemic heart disease (IHD). **Methods:** We analysed data from the National Registry of Heart Failure, a prospective study that included patients admitted for HF in Internal Medicine units nationwide. Patients with HFpEF were classified according to the use of statins, and the differences between the two groups were analysed. A multivariable analysis was performed using Cox regression to assess factors independently related to mortality. **Results:** A total of 2788 patients with HFpEF were included; 63% of them were women with a mean age of 80.1 (±7.8) years. The statin-treated group (40.2%) was younger, with better functional status, and had a more common diagnosis of vascular disease and lower frequency of atrial fibrillation. The most frequent aetiology of HF in both groups was the hypertensive one. Nevertheless, ischaemic HF was more common in those who received statins (24.8% vs. 9.6%; *p* < 0.001). Multivariable analysis showed lower mortality at the 1-year follow-up in statin-treated patients (OR: 0.74; 95%CI: 0.61–0.89; *p* = 0.002). This association was observed in patients without IHD (*p* < 0.001) but not in those with IHD (*p* = 0.11). **Conclusions:** Statins are associated with a decrease in total mortality in patients with HFpEF. This benefit occurs mainly in those without IHD.

## 1. Introduction

Heart failure (HF) is a leading cause of hospitalisation and death in people over 65 years of age and predominantly affects older patients [1,2]. This syndrome is classified according to left ventricular ejection fraction (EF) in HF with preserved EF (HFpEF) ≥ 50%), HF with reduced EF (HFrEF) (≤40%), and HF with mildly reduced ejection fraction (HFmrEF) (41–49%) [3]. HFpEF accounts for approximately half of all HF patients, and its prevalence is increasing [1]. Until recently, in this type of HF, in contrast to HFrEF, no drug has been shown to reduce mortality [1,2,3,4]. In fact, only sodium-glucose cotransporter type 2 inhibitors (SGLT2i) and finerenone have recently shown benefits. Dapagliflozin and Empagliflozin have demonstrated a reduction in the combined endpoint of readmissions and cardiovascular mortality in patients with HFpEF [5,6], and some meta-analyses have shown a decrease in isolated cardiovascular mortality, not in individual clinical trials [7,8]. On the other hand, in the FINEARTS-HF trial, finerenone reduced the combined primary end-point of HF and cardiovascular death in HFpEF [9]. Nevertheless, the effect of beta-blockers, aldosterone antagonists, angiotensin-converting enzyme inhibitors (ACE inhibitors), angiotensin II receptor antagonists (ARBs), and the angiotensin receptor neprilysin inhibitors (ARNi), digoxin and ivabradine, have been analysed without benefit [10].

Several observational studies have shown a reduction in mortality with the use of statins in patients with HFpEF [10,11,12,13,14,15,16,17,18,19,20,21,22,23,24,25,26]. Some of them suggest that this effect is independent of LDL cholesterol levels [11,17,26,27]. Benefit has even been found in patients with HFpEF of non-ischaemic aetiology [14,17,27]. It is also worth mentioning that several meta-analyses support a decrease in mortality in patients with HFpEF taking statins [11,22,28,29]. Nevertheless, the efficacy of statins in patients with HFrEF outside the indication of coronary heart disease has not been demonstrated, as was found in the CORONA (Controlled Rosuvastatin Multinational Trial in Heart Failure) clinical trial, in which rosuvastatin did not reduce mortality, but did reduce hospitalisations due to cardiovascular causes [30]. Similarly, another clinical trial (GISSI trial) showed no decrease in mortality in patients treated with statins (Rosuvastatin). This was the only clinical trial conducted that analysed patients with HFrEF and HFpEF separately. However, only 10% of patients had HFpEF [31].

The lack of effective therapies in HFpEF and insufficient evidence on the benefit of statins without clinical trials in this type of HF [4] make it necessary to study the impact of these drugs, especially if they have a role in patients without ischaemic heart disease as some studies have suggested [17,27]. Statins may represent a change in the current management of these patients. Despite this, the latest HF guidelines state that statins are only indicated for coronary artery disease [4,32], probably due to limited evidence and some studies denying their benefit unless otherwise specifically indicated [23,31].

The purpose of this study is to analyse whether statin therapy improves the prognosis of patients with HFpEF and especially whether the effect differs according to the presence or absence of ischaemic heart disease.

## 2. Materials and Methods

### 2.1. Design

An observational study was conducted using data from the Spanish National Registry of Heart Failure (RICA). The RICA registry is a prospective multicentre cohort study with the aim of analysing the characteristics of patients admitted for HF in Internal Medicine units nationwide.

### 2.2. Population, Study Scope and Recruitment

This registry includes data from patients from 52 Spanish hospitals. These patients were over 50 years of age discharged after a hospitalisation due to HF (either debut or exacerbated chronic) and followed up for at least one year. All patients had to have an echocardiography to assess LVEF, and the diagnosis of HF was made according to the criteria of the European Society of Cardiology [3].

For this study, only patients with a diagnosis of HFpEF were included from March 2008 to September 2018. Patients with HF secondary to severe pulmonary hypertension, refusal to participate, patients currently participating in a clinical trial, or those who could not be followed up were excluded. The follow-up consists of visits after three months and one year. The readmissions and mortality were collected.

### 2.3. Study Variables

Socio-demographic characteristics (age, sex), height, weight, body mass index (BMI), functional capacity (assessed by the Barthel index [33]), and mental status by the Pfeiffer test [34] were collected.

Comorbidities were also included using the Charlson Index [35], as well as other comorbidities not included in this index, such as arterial hypertension, dyslipidaemia, ischaemic heart disease, atrial fibrillation, and anaemia. These diagnoses were extracted from hospital medical records.

Functional class according to the New York Heart Association (NYHA) classification and heart rate (HR) and some vital signs like systolic blood pressure (SBP) and diastolic blood pressure (DBP) were also recorded.

Some blood test data such as creatinine, haemoglobin, estimated glomerular filtration rate (eGFR), and N-terminal portion of B-type natriuretic peptide (NT-proBNP) were recorded. It also included the treatment prescribed at discharge: statins, beta-blockers, ACE inhibitors, ARBs, aldosterone antagonists, loop diuretics, thiazide diuretics, digoxin, and ivabradine.

### 2.4. Statistical Analysis

Initially, a descriptive study was carried out. Categorical variables were expressed as frequencies and percentages, and quantitative variables as mean and standard deviation (SD) or as median and interquartile range, depending on whether the distribution was normal or non-normal.

Subsequently, differences between patients regarding whether they received statin treatment or not were analysed. The Chi-square test or Fisher’s exact test was used to assess the relationship between categorical variables, and the Student’s *t*-test or Mann–Whitney U test for quantitative variables, depending on whether the variables followed a normal distribution or not. The by Kolmogorov–Smirnov test was used to determine whether quantitative variables were normally distributed. All-cause mortality after 1 year was analysed in both groups, treated or not with statins, and Kaplan–Meier curves were built to observe the prognostic differences between both groups. To assess whether statin use was independently associated with mortality, a multivariable analysis was performed using the Cox regression model, including variables which, in the univariable analysis, showed a statistically significant relation with the probability of death.

To assess whether the effect of statins differed in patients with and without ischaemic heart disease, mortality-related factors were analysed separately in both groups by univariable and multivariable Cox regression analysis. A *p*-value of less than 0.05 was considered statistically significant. The odds ratio (OR) and hazard ratio (HR) were used as a measure of the magnitude of association and were expressed together with their 95% confidence interval (95% CI). Statistical analysis was performed with SPSS software (Statistical Package for the Social Sciences, IBM Corp. IBM SPSS Statistics for Windows, Version 29.0, Armonk, NY, USA: IBM Corp).

### 2.5. Ethical Considerations

The RICA registry protocol conforms to the ethical guidelines of the Declaration of Helsinki. It was approved by the Ethics Committee of the Hospital Universitario Reina Sofía (Córdoba), and all patients signed the informed consent before being included in the RICA cohort. The registry protocol was initially approved by the Ethics Committee of the Hospital Universitario Reina Sofía de Córdoba and was subsequently approved by each of the committees of the participating hospitals (code 18/349-E, last updated on 9 August 2018). All patients signed an informed consent form prior to inclusion in the registry. The data were collected from a web page (www.registrorica.org, accessed on 1 January 2008) containing the anonymous database and accessed by each investigator through a personalised password.

## 3. Results

### 3.1. Descriptive Analysis

A total of 2788 patients with a diagnosis of HFpEF were included, of whom 1031 (37%) were male and 1757 (63%) female, with a mean age of 80.1 years ± SD: 7.8; range 50–100 years. The median overall follow-up time was 328.6 days, 340 days in statin-treated patients and 323.2 days in non-statin-treated patients.

### 3.2. Characteristics of Patients on Statin Therapy

Of the total, 1121 (40.2%) patients were taking statins and 1667 (59.8%) were not. The relationship between statin intake and demographic characteristics and comorbidities is detailed in Table 1.

Patients receiving statin treatment at discharge were younger (mean 79.4 vs. 80.5 years; *p* < 0.001), with no significant differences in sex compared to those who did not take statins.

Regarding medical history, statin-treated patients were significantly more likely to have hypertension, diabetes, dyslipidaemia, or obesity. In addition, they had more history of myocardial infarction, ischaemic stroke, and peripheral arterial disease. Conversely, they had less atrial fibrillation, dementia, and chronic obstructive pulmonary disease (COPD). Patients on statin therapy had a higher comorbidity as assessed by the Charlson index (mean 3.2 vs. 2.55; *p* < 0.001).

On the other hand, patients on statin treatment had a better functional and mental status with lower functional dependency according to the Barthel index and a Pfeiffer test with fewer errors (Table 1).

Regarding the aetiology of HF (Table 2), hypertension was the most frequent cause of HF in both groups, although it predominated, almost significantly, in patients without statins (51.5% vs. 47.7%; *p* = 0.053). Ischaemic aetiology was higher in statin-treated patients (24.8% vs. 9.6%; *p* < 0.001), while valvular aetiology (moderate-severe valvular heart disease) was less frequent in this group (16.6% vs. 22.7%; *p* < 0.001).

Statin-treated patients had a better NYHA functional class. There was no difference in the percentage of debut HF between the two groups.

Regarding pharmacological treatment at discharge (Table 2), statin-treated patients received more ACE inhibitors or ARBs, beta-blockers, loop diuretics, and ivabradine, while more non-statin-treated patients were treated with digoxin.

At the 1-year follow-up, patients receiving statins had more readmissions for any cause (41.8% vs. 37.8%; *p* = 0.030) and for HF (25.5% vs. 21%; *p* = 0.005). However, 1-year all-cause mortality was significantly lower in this group of patients (14.7% vs. 20.9%; *p* < 0.001).

### 3.3. Factors Related to Mortality

**Figure 1:**
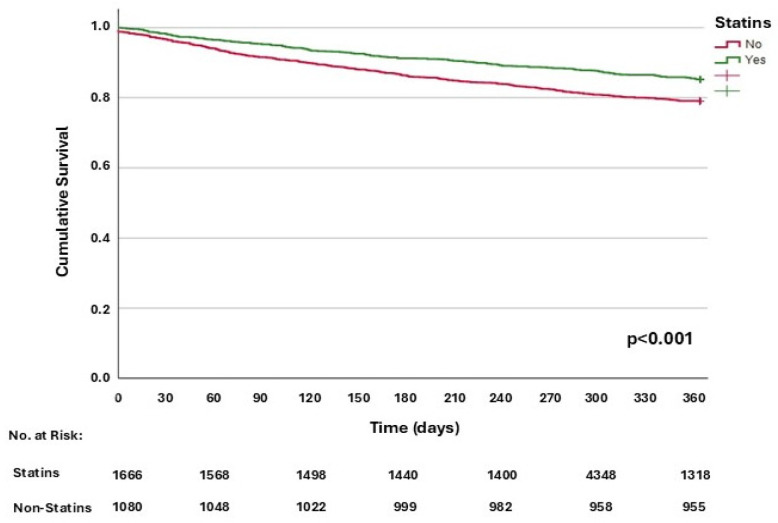
Kaplan–Meier function for one-year all-cause mortality, according to statin use.

In the univariable analysis (Table 3), female sex, obesity, systolic blood pressure, statin, ACE inhibitors or ARBs, and beta-blockers intake were significantly related to a lower mortality. On the other hand, age, presence of dementia, atrial fibrillation, COPD, neoplasia, anaemia, hyponatraemia, eGFR < 60 mL/min, ≤ 60 Barthel index, NYHA functional class III or IV, aldosterone antagonists, and digoxin intake were significantly associated with higher mortality. Figure 1 shows the Kaplan–Meier survival curves for one-year mortality based on whether or not patients are treated with statins.

In multivariable Cox regression analysis, statins were independently associated with lower 1-year mortality, as were female sex and obesity. In contrast, dementia, anaemia, eGFR <60 mL/min, hyponatraemia, NYHA classes III–IV, functional impairment (Barthel Index ≤ 60), aldosterone antagonists, and digoxin were independent predictors of mortality (Table 3).

**Figure 2:**
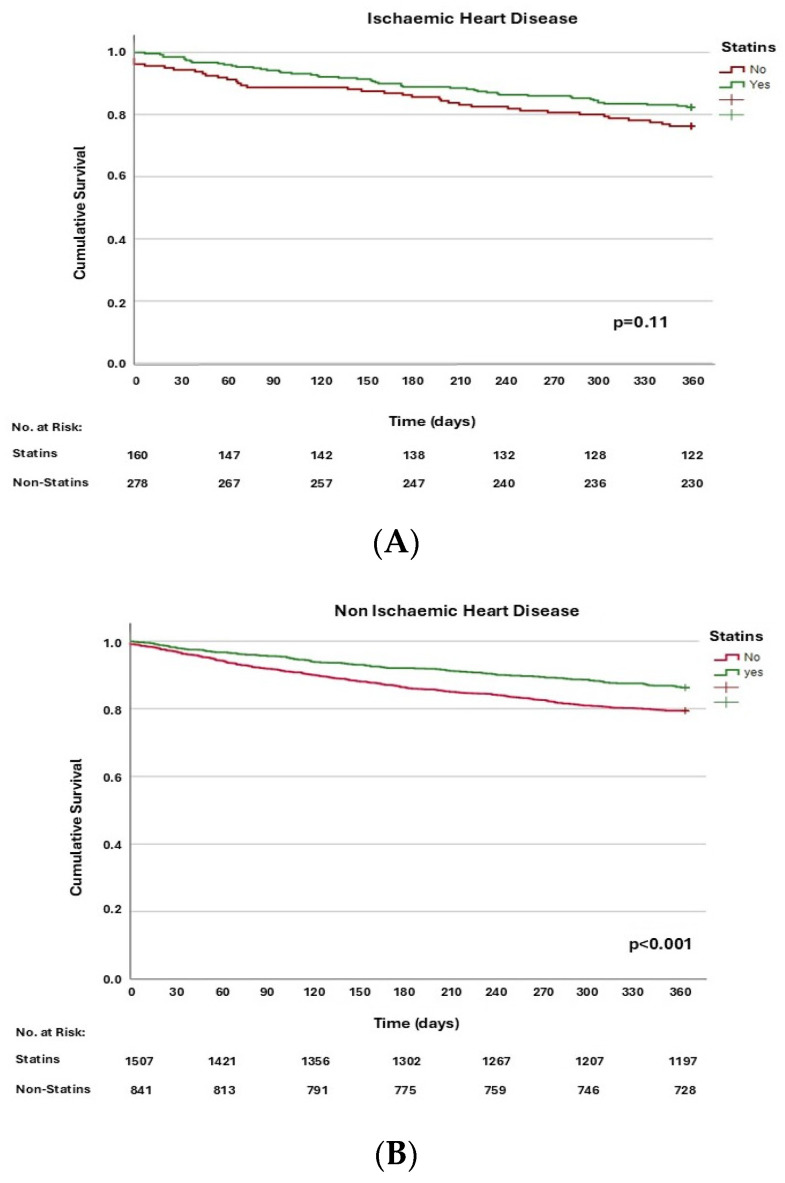
Kaplan–Meier function for one-year all-cause mortality, according to statin use in patients with ischaemic heart disease (**A**) and without ischaemic heart disease (**B**).

When analysing separately whether statins influenced mortality in patients with or without ischaemic heart disease (Table 4), it was observed that, in patients without ischaemic heart disease, statin use was independently associated with reduced mortality (OR: 0.69; 95% CI: 0.56–0.86; *p* < 0.001). However, in patients with HFpEF and ischaemic heart disease, there was no association between statin treatment and mortality (OR: 0.69; 95% CI: 0.43–1.11; *p* = 0.110). Kaplan–Meier curves for one-year all-cause mortality according to statin use in patients with and without ischaemic heart disease are represented in Figure 2A and Figure 2B, respectively.

## 4. Discussion

This study shows that statin therapy is independently associated with lower mortality in patients with HFpEF, primarily in patients without ischaemic heart disease.

Patients receiving statins had more vascular risk factors and related comorbidities. These results are to be expected, given the indication of statins for the treatment of hypercholesterolaemia and for primary and secondary prevention of vascular disease [36,37].

In addition, the fact that statin-treated patients had better functional and mental status may be related to the higher prescription of these drugs because of the longer life expectancy.

The independent association between statins and lower mortality in HF patients has been previously described in several studies, most of them observational [10,11,12,13,14,15,16,17,18,19,20,21,22,23,24,25,26,27,28,38]. The *GISSI* study was the only clinical trial conducted that included patients with HFrEF and HFpEF [31], analysing each group separately. It did not show a significant decrease in mortality. However, the low percentage of patients with HFpEF in the study (10%) may have precluded the assessment of the effect of statins in this subgroup. In addition, patients with mildly reduced LVEF were included in the HFpEF group (HFpEF was defined as those with LVEF greater than 40%), which has been shown to have more similar characteristics to HFrEF [4]. A minority of studies have shown no reduction in mortality [12,24,25]. However, there have been several meta-analyses [11,22,28,29], one of them published recently [28], showing the benefit of statin use, given its association with a reduction in total mortality in patients with HFpEF.

Most previous studies have assessed the reduction in overall mortality, and some of them have also assessed cardiovascular mortality. Notably, it has been suggested that the mortality benefit is mainly due to a reduction in sudden death and non-cardiovascular death [12]. In addition, mortality reduction has been reported in patients without ischaemic heart disease [14,17,27]. Specifically, a subanalysis of the TOPCAT clinical trial found a reduction in all-cause and cardiovascular mortality among patients who did not have ischaemic heart disease and were taking statins compared to those who were not taking statins. This effect was not observed in patients with ischaemic heart disease [17]. These results are consistent with those observed in our study. Furthermore, another recent observational study showed that statins reduced mortality and cardiovascular events separately. An interesting aspect of this study is that it had a large sample of patients, and those with cardiovascular disease were excluded. Thus, statins were assessed only as primary prevention [39].

The pathophysiology of HFpEF is complex and different from HFrEF [40,41]. Treatments such as ACE inhibitors, ARBs, beta-blockers, and aldosterone antagonists are part of the optimal therapy in patients with HFrEF because of their benefit in reducing mortality, which has not been achieved in patients with HFpEF [24]. This supports the premise that they are differentiated groups of patients and contributes to the understanding that a pharmacological group may be useful for one type of HF but not for the other, as may be the case with statins [30,42]. These drugs, in addition to their widely known action on LDL-cholesterol levels, are postulated to have numerous effects related to the development and progression of HFpEF [43]. These include beneficial effects on ventricular remodelling, with reduced left ventricular hypertrophy and fibrosis and prevention of left ventricular dilatation in both animal models and patients [44,45]. A mild antihypertensive effect is added in hypertensive patients [46], as well as an improvement in arterial distensibility due to improved endothelial function and reduced atherosclerosis with plaque stabilisation [47], thus reducing afterload and improving coronary perfusion. These results imply an improvement in left ventricular relaxation and diastolic function [48].

These effects are also associated with a decrease in the frequency of atrial fibrillation development [49]. Indeed, in our study, a lower prevalence of atrial fibrillation was observed in patients treated with statins. It is also thought to reduce ventricular tachyarrhythmias, both through its “anti-remodelling” effect and its effects on microcirculation and ischaemia, as well as by normalising sympathetic innervation, which may benefit those with excess catecholaminergic activity. In particular, it has been associated with reduced QT interval variability, QT shortening and increased pulse variability [50]. This may be implicated in the reduction of sudden death in these patients [20]. In addition, it is believed that its benefits may be related to its anti-inflammatory and antioxidant capacity, causing a decrease in analytical parameters such as C-reactive protein (CRP) or brain natriuretic peptide (BNP), which is advantageous considering the involvement of systemic inflammation in the pathophysiology of HFpEF [28,40]. Some authors consider that many patients with HFpEF have subclinical ischaemia, and even if they do not have macrovascular ischaemic disease demonstrated by events or angiography, statins have a benefit at that level [21,42].

Indeed, as mentioned at the outset, our study shows that the mortality benefit of statins occurs in patients without ischaemic heart disease, which supports that it is not only due to their lipid-lowering effect. Other studies also support this position [11,17,22,27].

It should be noted that the decrease in mortality of statins in these patients could be associated with their benefits in other comorbidities (renal failure, diabetes, infections) [12,36], in addition to the pure vascular effects mentioned above.

In addition to statins, other factors independently related to lower mortality were female sex and obesity. While worse NYHA functional status, the presence of renal disease, anaemia, hyponatraemia, and dementia were independently associated with higher mortality. This could be expected given that these are poor prognostic factors that are widely described in the literature [2,41].

Interestingly, aldosterone antagonists were associated with an increase in mortality in multivariable analysis. Nevertheless, in a sub-analysis of the TOPCAT study [51] in an American population, spironolactone does reduce mortality as an isolated variable. In the FINEARTS-HF trial [9], finerenone decreases the combined event of readmissions and mortality but does not reduce mortality in isolation. Other studies demonstrate their lack of effectiveness on survival in HFpEF [24]. In our study, they are also associated with increased mortality, which may be due to their use in patients who are more refractory to treatment, with more comorbidities, or to the adverse effects of the medication. Digoxin was also associated with increased mortality, probably due to its association with the diagnosis of atrial fibrillation and the comorbidity that this entails in patients with HFpEF.

On the other hand, statin-treated patients had more readmissions overall and for HF, reduced readmissions with statin use have been reported in the literature [13,42], although not unanimously [12]. The higher number of admissions of these patients could be related to the decrease in mortality.

Several observational studies claim that some of these effects are ineffective after established cardiac hypertrophy or dilatation or high NYHA functional class; specifically, the “anti-remodelling” effect and symptom-reducing effect [12,26]. These findings may indicate the importance of early treatment of patients with incipient or non-advanced HFpEF to prevent, delay or reduce the deleterious effects related to cardiac remodelling. This may imply considering the possibility of a lower success rate in patients with advanced heart disease, with the consequent likelihood of a higher number of treatment-related adverse effects.

On the other hand, some studies have found that low cholesterol levels are associated with increased mortality [52]. This could be related to the advanced stage of HF and consequent secondary malnutrition, in the same sense that lower BMI is associated with increased mortality, a phenomenon known as the “obesity paradox” [53]. Such a result is observed in our study, as patients with obesity have lower mortality after adjusting for other risk factors.

This study has several limitations. First, it is an observational study, which does not allow us to attribute causality. In addition, residual confounding factors may exist. However, a large sample of patients was collected from multiple hospitals nationwide.

Secondly, cholesterol levels were not collected, making it difficult to assess whether the effect of statins is independent of cholesterol reduction.

Thirdly, neither the type of statins (low or high intensity, lipophilic, or hydrophilic) nor the dose or duration of treatment was collected. Therefore, despite evidence of a greater effect of lipophilic and high-intensity statins [11,26,28], we were unable to analyse these data. However, most studies do not have these data available either [29].

In addition, underuse of statins was observed in the non-statins treated cohort, despite their strong indication in patients with established cardiovascular disease.

Moreover, the echocardiographic data included in the study were limited, which restricted the ability to fully explore possible cardiac structural or functional differences between the groups. 

Finally, there were also significant variations in baseline characteristics of the two cohorts, which is an important factor to consider when interpreting the association found in the study.

As previous studies indicate, clinical trials are needed to definitively establish the association between statin use and lower mortality in patients with HFpEF. This is particularly relevant in this pathology, where only one pharmacological group has been shown to reduce mortality. Although the available evidence comes from observational studies, several meta-analyses highlight the potential benefit of reducing mortality [11,22,28,29]. Therefore, a mention of the potential benefit of statin use in patients with HFpEF should be considered for inclusion in HF clinical practice guidelines, and at least explicit mention should be made of the main studies and meta-analyses showing these results, although this does not mean that it is justified to routinely recommend their use. In fact, we cannot routinely recommend statins in HF-pEF because we lack prospective randomised trials. However, the benefit of statins is well established in secondary and in primary prevention of cardiovascular events. We could recommend close monitoring of LDL-cholesterol levels to initiate statin therapy when indicated, taking into account other cardiovascular risk factors and the potential benefit in these patients with HFpEF.

## 5. Conclusions

Statin use is independently associated with lower 1-year mortality in patients with HFpEF, particularly those without ischaemic heart disease. However, it does not correlate with reduced rates of total or heart failure-specific readmissions. These findings, together with the current evidence, suggest that in the future, statins could form part of guidelines as a beneficial strategy in HFpEF. However, more prospective randomised trials are needed.

## Figures and Tables

**Table 1:** Demographic characteristics, comorbidities, and scales of assessment of patients with HFpEF according to whether they were taking statins or not.

	Total N = 2788	Statin-Treated N = 1121 (40.2%)	Non-Statin-Treated N = 1667 (59.8%)	*p*	OR (95% CI)
Demographics					
Age (years).*	80.1 ± 7.8	79.4 ± 7.6	80.5 ± 8	**<0.001**	0.98 (0.97–0.99)
Sex: Women, ** Men, **	1757 (63) 1031 (37)	703 (62.7) 418 (37.3)	1054 (63.2) 613 (36.8)	0.782	0.97 (0.84–1.14)
Comorbidities					
Hypertension, **	2470 (88.6)	1040 (92.8)	1430 (85.8)	**<0.001**	2.13 (1.63–2.77)
Diabetes, **	1255 (45)	631 (56.3)	624 (37.4)	**<0.001**	0.47 (0.40–0.54)
Dyslipidaemia, **	1368 (49.1)	921 (82.2)	447 (26.8)	**<0.001**	0.08 (0.07–0.09)
Obesity (BMI > 30), **	1204 (43.2)	520 (46.4)	684 (41)	**0.005**	1.24 (1.07–1.45)
Myocardial infarction, **	465 (16.7)	308 (27.5)	157 (9.4)	**<0.001**	0.27 (0.22–0.34)
Stroke, **	370 (13.3)	189 (16.9)	181 (10.9)	**<0.001**	0.60 (0.48–0.75)
Peripheral arterial disease, **	250 (9)	127 (11.3)	123 (7.3)	**<0.001**	0.62 (0.48–0.81)
Atrial fibrillation (ECG), **	1699 (60.9)	626 (55.8)	1073 (64.6)	**<0.001**	0.70 (0.60–0.82)
COPD, **	651 (23.4)	236 (21.1)	415 (24.9)	**0.019**	1.24 (1.04–1.49)
Dementia, **	141 (5.1)	42 (3.7)	99 (5.9)	**0.010**	1.62 (1.12–2.35)
Neoplasia, **	311 (11.2)	115 (10.3)	196 (11.8)	0.218	1.17 (0.91–1.49)
Anaemia, **	1613 (57.9)	667 (59.5)	946 (56.7)	0.149	1.12 (0.96–1.31)
eGFR < 60 mL/min, **	1624 (58.2)	678 (60.5)	946 (56.7)	0.050	1.17 (1–1.36)
Hyponatraemia, **	424 (15.2)	171 (15.3)	253 (15.2)	0.956	1 (0.81–1.24)
Scales of assessment					
Charlson Index, *	2.8 ± 2.4	3.2 ± 2.4	2.55 ± 2.3	**<0.001**	1.12 (1.08–1.16)
Barthel Index, *	80.6 ± 23	82.4 ± 21.9	79.4 ± 23.7	**<0.001**	1.006 (1.002–1.009)
Barthel Index < 60, **	470 (16.9)	161 (14.4)	309 (18.5)	**0.004**	0.74 (0.60–0.91)
Pfeiffer Test, *	1.63 ± 2	1.5 ± 1.8	1.7 ± 2.1	**0.003**	0.94 (0.91–0.98)
Pfeiffer Test ≥ 3 wrong, **	628 (25.4)	237 (22.8)	391 (27.2)	**0.012**	0.79 (0.65–0.95)

Legend: Categorical variables are expressed in number and percentage: ** n (%). Quantitative variables are expressed as * mean ± standard deviation (SD). OR: odds ratio; 95% CI: 95% confidence interval; BMI: body mass index; ECG: electrocardiogram; COPD: chronic obstructive pulmonary disease; eGFR: estimated glomerular filtration rate.

**Table 2:** Characteristics of heart disease, vital signs, blood test data, treatment at discharge, and outcome (deaths and readmissions at one year) of patients with HFpEF according to the intake of statins.

	Total N = 2788	Statin-Treated N = 1121 (40.2%)	Non-Statin-Treated N = 1667 (59.8%)	*p*	OR (95% CI)
LVEF (%), *	61.8 ± 8.1	61.3 ± 8.3	62.1 ± 7.8	**0.014**	0.988 (0.98–0.99)
LAD (mm)	47 (41–52)	46(41–51)	48 (42–53)	**<0.001**	1.012 (1.001–1.023)
PASP (mmHg)	45 (37–56)	45 (35–54.5)	46 (38–58)	**0.010**	1.013 (1.006–1.019)
LVESD (mm)	31 (26–37)	31 (26–37)	30.5 (26–37)	0.263	0.997 (0.989–1.005)
LVEDD (mm)	48 (42–53)	48 (42.5–53)	47.3 (42–52)	0.251	0.996 (0.987–1.004)
NYHA: I–II, ** III–IV, **	1782 (65.1) 954 (34.9)	750 (67.4) 363 (32.6)	1032 (63.6) 591 (36.4)	**0.040**	0.85 (0.72–0.99)
debut HF, **	928 (33.3)	367 (32.7)	561 (33.7)	0.615	0.96 (0.82–1.13)
Aetiology of HF					
Hypertensive **	1393 (50)	535 (47.7)	858 (51.5)	0.053	0.86 (0.74–1)
Ischaemic, **	438 (15.7)	278 (24.8)	160 (9.6)	**<0.001**	3.12 (2.51–3.84)
Valvular, **	565 (20.3)	186 (16.6)	379 (22.7)	**<0.001**	0.68 (0.56–0.82)
Hypertrophic, **	51 (1.8)	18 (1.6)	33 (2)	0.470	0.81 (0.45–1.44)
Alcoholic, **	10 (0.4)	3 (0.3)	7 (0.4)	0.510	0.64 (0.16–2.47)
Non-affiliated, **	164 (5.9)	50 (4.5)	114 (6.8)	**0.009**	0.64 (0.45–0.90)
Others, **,†	147 (5.3)	39 (3.5)	108 (6.5)	**0.001**	0.52 (0.36–0.76)
Vital signs					
SBP (mmHg), *	140.3 ± 27.1	142.2 ± 27.4	139 ± 26.8	**0.002**	1.004 (1.002–1.007)
DBP (mmHg), *	75 ± 15.9	74.8 ± 15.6	75.1 ± 16	0.590	0.99 (0.992–0.996)
HR (lpm), *	86.1 ± 22.3	84.8 ± 21.4	86.9 ± 22.8	**0.015**	0.999 (0.994–1.003)
Blood test data					
Cr (mg/dL), *	1.3 ± 0.7	1.3 ± 0.7	1.3 ± 0.7	0.065	1.11 (0.99–1.24)
Hb (g/dL), *	11.9 ± 2	11.8 ± 2	11.9 ± 2	0.271	0.98 (0.94–1.02)
NT-proBNP (pg/mL), ***	2954 [1429.5–6343.5]	2831 [1330–6481]	3096 [1461–6304.5]	0.469	1.01 (0.99–1.03)
C-reactive protein (mg/L) ***	5.9 [1.5–17.2]	5.5 [1.5–18.7]	6.3 [1.6–17]	0.865	1.000 (0.997–1.003)
Discharge treatment					
ACE inh., **	994 (35.7)	413 (36.8)	581 (34.9)	0.282	1.09 (0.93–1.28)
ARBs, **	838 (30.1)	401 (35.8)	437 (26.2)	**<0.001**	1.57 (1.33–1.85)
ACE inh. or ARBs, **	1809 (64.9)	804 (71.7)	1005 (60.3)	**<0.001**	1.67 (1.42–1.97)
Beta-blockers, **	1437 (51.5)	671 (59.9)	766 (46)	**<0.001**	1.75 (1.50–2.05)
Loop diuretics, **	2383 (85.5)	1012 (90.3)	1371 (82.2)	**<0.001**	2.00 (1.59–2.53)
Aldosterone antagonists, **	621 (22.3)	254 (22.7)	367 (22)	0.689	1.04 (0.86–1.24)
Thiazide diuretics, **	290 (10.4)	123 (11)	167 (10)	0.418	0.90 (0.71–1.16)
Ivabradine, **	28 (1)	20 (1.8)	8 (0.5)	**0.001**	3.77 (1.65–8.58)
Digoxin, **	550 (19.7)	180 (16.1)	370 (22.2)	**<0.001**	0.67 (0.55–0.82)
Deaths and readmissions after 1 year					
Deaths, **	514 (18.4)	165 (14.7)	349 (20.9)	**<0.001**	0.65 (0.53–0.80)
Readmissions, **	1098 (39.4)	468 (41.8)	630 (37.8)	**0.033**	0.85 (0.72–0.99)
Readmissions for HF, **	636 (22.8)	286 (25.5)	350 (21)	**0.005**	0.78 (0.65–0.93)

Legend: Categorical variables are expressed in number and percentage: ** n (%). Quantitative variables are expressed as * mean ± standard deviation (SD), *** or as median [interquartile range]. OR: odds ratio; 95% CI: 95% confidence interval; LVEF: left ventricle ejection fraction; LA: left atrial diameter; PASP: pulmonary artery systolic pressure; LVESD: left ventricular end-systolic diameter; LVEDD: left ventricular end-diastolic diameter; NYHA: Functional Classification of the New York Heart Association; HF: heart failure; SBP: systolic blood pressure; DBP: diastolic blood pressure; HR: heart rate; Cr: creatinine; Hb: haemoglobin; NT-proBNP: N-terminal portion of B-type natriuretic peptide; ACE inh.: angiotensin-converting enzyme inhibitors; ARBs: angiotensin receptor blocker. † Other aetiologies include infiltrative, arrythmias or pericardial diseases.

**Table 3:** Univariable and multivariable analysis (Cox Regression) of factors related to overall mortality.

	Overall			
	Univariable Analysis		Multivariable Analysis	
	OR (95% CI)	*p*	HR (95% CI)	*p*
Age	1.04 (1.03–1.06)	**<0.001**	1.02 (1.01–1.04)	**<0.001**
Women	0.79 (0.65–0.97)	**0.02**	0.72 (0.59–0.87)	**<0.001**
Hypertension	0.97 (0.72–1.31)	0.83	___	
Diabetes	1.05 (0.87–1.23)	0.59	___	
Dyslipidaemia	1.11 (0.92–1.35)	0.27	___	
Obesity (BMI > 30)	0.70 (0.58–0.86)	**0.001**	0.76 (0.64–0.94)	**0.008**
Myocardial infarction	1.15 (0.89–1.47)	0.28	___	
Atrial fibrillation	1.28 (1.05–1.56)	**0.017**	0.99 (0.82–1.21)	0.945
COPD	0.77 (0.62–9.95)	**0.015**	1.13 (0.92–1.40)	0.246
Dementia	2.17 (1.51–3.15)	**0.009**	1.53 (1.11–2.11)	**0.009**
Neoplasia	1.51 (1.14–1.99)	**0.004**	1.22 (0.95–1.57)	0.129
Anaemia	1.59 (1.30–1.94)	**<0.001**	1.28 (1.06–1.55)	**0.011**
eGFR < 60 mL/min/1.73 m^2^	1.71 (1.40–2.10)	**<0.001**	1.57 (1.30–1.91)	**<0.001**
Hiyponatraemia	1.48 (1.15–1.89)	**0.002**	1.26 (1.01–1.58)	**0.040**
Barthel Index ≤ 60	2.36 (1.88–2.95)	**<0.001**	1.77 (1.43–2.18)	**<0.001**
NYHA (III–IV)	1.90 (1.56–2.31)	**<0.001**	1.56 (1.30–1.87)	**<0.001**
SBP	0.99 (0.990–0.997)	**0.001**	0.99 (0.994–1.001)	0.223
HR	0.997 (0.99–1.00)	0.14	___	
ACE inh o ARBs	0.78 (0.64–0.95)	**0.012**	0.93 (0.78–1.12)	0.465
Beta-blockers	0.82 (0.67–0.98)	**0.032**	0.96 (0.81–1.16)	0.695
Aldosterone antagonists	1.30 (1.05–1.61)	**<0.001**	1.34 (1.10–1.62)	**0.004**
Digoxin	1.40 (1.11–1.76)	**0.004**	1.34 (1.08–1.66)	**0.007**
**Statins**	**0.65 (0.53–0.80)**	**<0.001**	**0.74 (0.61–0.89)**	**0.002**

Legend: OR: Odds ratio; HR: Hazard ratio; 95% CI: 95% confidence Interval. BMI: body mass index; COPD: chronic obstructive pulmonary disease; eGFR: estimated glomerular filtration rate; NYHA: Functional Classification of the New York Heart Association; SBP: systolic blood pressure; HR: heart rate; ACE inh.: angiotensin-converting enzyme inhibitors; ARBs: angiotensin receptor blocker.

**Table 4:** Univariable and multivariable analysis of factors related to mortality in patients without and with ischaemic heart disease.

	Without Ischaemic Heart Disease				With Ischaemic Heart Disease			
	Univariable Analysis		Multivariable Analysis		Univariable Analysis		Multivariable Analysis	
	OR (CI 95%)	*p*	HR (CI 95%)	*p*	OR (CI 95%)	*p*	HR (CI 95%)	*p*
Age	1.05 (1.03–1.07)	**<0.001**	1.03 (1.02–1.04)	**<0.001**	1.01 (0.98–1.01)	0.500	1.00 (0.97–1.04)	0.861
Women	0.81 (0.66–1.01)	0.061	0.76 (0.61–0.94)	**0.012**	0.74 (0.46–1.18)	0.200	0.69 (0.44–1.07)	0.098
Hypertension	1.02 (0.74–1.41)	0.910	___		0.69 (0.32–1.48)	0.340	___	
Diabetes	0.96 (0.77–1.18)	0.680	___		0.87 (0.54–1.40)	0.550	___	
Dyslipidaemia	0.84 (0.69–1.05)	0.122	___		1.14 (0.70–1.88)	0.620	___	
Obesity (BMI > 30)	0.70 (0.56–0.86)	**0.001**	0.77 (0.63–0.95)	**0.013**	0.78 (0.48–1.28)	0.330	___	
Atrial fibrillation	1.38 (1.10–1.72)	**0.006**	1.03 (0.83–1.28)	0.788	0.99 (0.62–1.59)	0.980	___	
COPD	1.29 (1.01–1.63)	**0.038**	1.16 (0.92–1.46)	0.214	1.41 (0.83–2.39)	0.200	___	
Dementia	2.07 (1.38–3.11)	**<0.001**	1.40 (0.98–2.00)	0.069	2.77 (1.16–6.65)	**0.017**	2.17 (1.07–4.41)	**0.032**
Neoplasia	1.42 (1.06–1.95)	**0.019**	1.19 (0.90–1.56)	0.228	1.91 (0.97–3.77)	0.057	___	
Anaemia	1.70 (1.36–2.11)	**<0.001**	1.28 (1.04–1.58)	**0.020**	1.09 (0.67–1.79)	0.730	___	
eGFR < 60 mL/min/1.73 m^2^	1.84 (1.47–2.31)	**<0.001**	1.67 (1.35–2.07)	**<0.001**	1.17 (0.72–1.91)	0.530	___	
Hiyponatraemia	1.47 (1.11–1.92)	**0.006**	1.23 (0.96–1.57)	0.102	1.54 (0.84–2.79)	0.160	___	
Barthel Index ≤ 60	2.39 (1.86–3.06)	**<0.001**	1.82 (1.45–2.29)	**<0.001**	2.20 (1.28–3.77)	**0.004**	1.66 (0.99–2.78)	0.054
NYHA (III–IV)	1.97 (1.59–2.44)	**<0.001**	1.52 (1.25–1.87)	**<0.001**	1.58 (0.97–2.57)	0.060	1.45 (0.92–2.23)	0.108
SBP	0.992 (0.988–0.996)	**<0.001**	0.99 (0.993–1.001)	0.165	0.999 (0.991–1.007)	0.780	___	
HR	0.993 (0.988–0.998)	**0.008**	0.99 (0.992–1.001)	0.139	1.015 (1.004–1.026)	**0.007**		
ACE inh o ARBs	0.79 (0.64–0.98)	**0.032**	0.98 (0.80–1.19)	0.811	1.015 (1.004–1.026)	**0.007**		
Beta-blockers	0.86 (0.70–1.06)	0.160	___		0.53 (0.33–0.87)	**0.010**	0.64 (0.41–0.99)	**0.047**
Aldosterone antagonists	1.83 (1.45–2.31)	**<0.001**	1.50 (1.22–1.85)	**<0.001**	0.69 (0.38–1.26)	0.230	___	
Digoxin	1.40 (1.10–1.79)	**0.006**	1.35 (1.08–1.71)	**0.010**	1.53 (0.78–3.04)	0.210	___	
**Statins**	**0.61 (0.49–0.77)**	**<0.001**	**0.69 (0.56–0.86)**	**<0.001**	0.69 (0.43–1.11)	0.110	___	

Legend: OR: odds ratio; HR: hazard ratio; 95% CI: 95% confidence Interval. BMI: body mass index; COPD: chronic obstructive pulmonary disease; eGFR: estimated glomerular filtration rate; NYHA: Functional Classification of the New York Heart Association; SBP: systolic blood pressure; HR: heart rate; ACE inh.: angiotensin-converting enzyme inhibitors; ARBs: angiotensin receptor blocker.

## Data Availability

The data presented in this study are available on request from the corresponding author. The data are not publicly available due to privacy restrictions.

## Appendix A. RICA Registry Members

Álvarez Rocha P, Anarte L, Aramburu-Bodas O, Arévalo-Lorido JC, Cabanes-Hernández Y, Carrascosa S, Carrera-Izquierdo M, Casado-Cerrada J, Conde-Martel A, Chivite D, Díez-Manglano J, Epelde F, Formiga F, GarcíaEscrivá D, Gómez-del Olmo V, González-Franco A, Llacer P, López-Castellanos, G, Manzano L, Martín-Ezquerro A, Montero-Pérez-Barquero M, Moreno-García MC, Muela A, Ormaechea G, Pérez-Calvo JI, Pérez-Silvestre J, Quirós-López R, Romero M, Ruíz-Ortega R, Satué-Bartolomé JA, Soler-Rangel L, Suárez-Pedreira I, Trullàs JC.
